# RNA interference-mediated silencing of DNA methyltransferase 1 attenuates neuropathic pain by accelerating microglia M2 polarization

**DOI:** 10.1186/s12883-022-02860-6

**Published:** 2022-10-01

**Authors:** Ying Tan, Zongjiang Wang, Tao Liu, Peng Gao, Shitao Xu, Lei Tan

**Affiliations:** 1 Department of Spinal Surgery, Weifang Traditional Chinese Medicine Hospital, No.1055, Weizhou Road, Kuiwen District, Weifang, 261041 China; 2 Department of Spinal Surgery, Sunshine Union Hospital, Weifang, 261041 China

**Keywords:** Neuropathic pain, RNA interference, DNA methyltransferase 1, Chronic constriction injury, Microglia, M2 polarization, PI3K/Akt pathway

## Abstract

**Background:**

DNA methyltransferase 1 (DNMT1) exerts imperative functions in neuropathic pain (NP). This study explored the action of RNA interference-mediated DNMT1 silencing in NP by regulating microglial M2 polarization.

**Methods:**

NP rat models were established using chronic constriction injury (CCI) and highly aggressive proliferating immortalized (HAPI) microglia were treated with lipopolysaccharide (LPS) to induce microglia M1 polarization, followed by treatment of DNMT1 siRNA or si-DNMT1/oe-DNMT1, respectively. The pain threshold of CCI rats was assessed by determining mechanical withdrawal threshold (MWT) and thermal withdrawal latency (TWL). Levels of inflammatory factors (TNF-α/IL-1β/IL-6/IL-10) and DNMT1 in rat L4-L6 spinal cord samples and HAPI cells were measured using ELISA, RT-qPCR, and Western blot. iNOS and Arg-1 mRNA levels were measured via RT-qPCR. DNMT1, M1 marker (iNOS), and M2 marker (Arg-1) levels in microglia of CCI rats were detected by immunofluorescence. Percentages of M1 microglia phenotype (CD16) and M2 microglia phenotype (CD206) were detected by flow cytometry. The phosphorylation of PI3K/Akt pathway-related proteins was determined by Western blot.

**Results:**

CCI rats exhibited diminished MWT and TWL values, increased pro-inflammatory cytokines, and decreased anti-inflammatory cytokine IL-10. Additionally, DNMT1 was upregulated in CCI rat microglia. DNMT1 siRNA alleviated CCI-induced NP and facilitated M2 polarization of microglia in CCI rats. DNMT1 knockdown inhibited LPS-induced M1 polarization of HAPI cells and promoted M2 polarization by blocking the PI3K/Akt pathway, but DNMT1 overexpression inhibited the M1-to-M2 polarization of microglia.

**Conclusion:**

RNA interference-mediated DNMT1 silencing accelerates microglia M2 polarization by impeding the PI3K/Akt pathway, thereby alleviating CCI-induced NP.

**Supplementary Information:**

The online version contains supplementary material available at 10.1186/s12883-022-02860-6.

## Introduction

Neuropathic pain (NP) is recently defined as pain resulting from a disease or lesion affecting the somatosensory nervous system by the International Association for the Study of Pain, afflicting approximately 10% of the total population [1, 2]. It is usually attributed to damage and pathological changes in the peripheral or central nervous system (CNS), manifested as abnormal pain, hyperalgesia, spontaneous pain, and paresthesia [3]. Additionally, the primary disease mainly includes nerve injury caused by trauma, autoimmune disorders, diabetes, cancer, infection, and chemotherapy [4]. Unfortunately, considerable patients are refractory to conventional medical treatment [5]. Due to the complexity of its etiology, the pathogenesis of NP has not been completely elucidated and has emerged as a challenging issue in clinical practice.

It is noteworthy that neuroinflammation exerts a vital role in NP development [6]. Microglia, as the main innate immune cell in the CNS, constitute an immunoprotective barrier against various injuries to the CNS [7]. Notably, microglia are considered primary mediators in neuroinflammation [8], affect brain development, maintain the neural environment, and respond to nerve injury and repair [9]. The activation of microglia is intensely linked with NP initiation [10], and inhibiting over-activated microglia can weaken NP, suggesting that microglia probably are potential therapeutic targets against NP [11]. Under stress conditions, microglia are activated and polarized into M1 or M2 types [12]. The M1 phenotype is featured by highly-upregulated inflammatory factors, such as tumor necrosis factor-α (TNF-α), interleukin (IL)-1β, IL-12, and inducible nitric oxide synthase (iNOS), causing neuroinflammatory responses; and conversely, the M2 phenotype is marked by the molecular features of CD206 and arginase (Arg-1) and releases beneficial mediators, such as IL-10 and transforming growth factor-β, which exerts a neuroprotective role [12–14]. Intriguingly, the M2 polarization of microglia could mitigate Alzheimer’s disease-related NP [15]. Additionally, dihydromyricetin extenuates NP by inducing the shift from M1 to M2 polarization by potentiating the aldehyde dehydrogenase 2 (ALDH2) activity [16]. Botulinum toxin type A facilitates microglia M2 polarization by repressing the purinergic ligand-gated ion channel 7 receptor (P2X7R), thus inhibiting chronic constriction injury (CCI)-elicited NP [17]. The compelling evidence has indicated the involvement of microglia polarization in NP. Therefore, the polarization switching from M1 to M2 phenotype is a promising treatment strategy for NP.

DNA methylation is induced by DNA methyltransferases (DNMTs), including DNMT1, DNMT3a, and DNMT3b [18], in which DNMT1 is vital in numerous processes, such as methylation maintenance, chromatin stability, and gene regulation [19]. Aberrant DNA methylation is presumably highly involved in CCI-induced NP [20]. DNMT1 and DNMT3a contribute to NP onset by epigenetically suppressing *Kcna2* in primary afferent neurons [21, 22]. DNMT1 is upregulated in the spared nerve injury-induced NP rat models [23], and inhibition of DNMT1 potentiates M2 polarization of microglia in Alzheimer’s disease (AD)-related NP [15]. DNMT inhibitors include genistein, 5-azacytidine (5-Aza), and N-phthalyl-L-tryptophan (RG108) [24–26]. Specifically, 5-Aza has an anti-inflammatory efficacy and can treat inflammatory lung injury [27]. Additionally, oral azacitidine (CC-486), a DNMT inhibitor, can be used to treat tumors [28]. However, DNMT inhibitors show certain limitations, such as the inherent cytotoxic effects of nucleoside DNMT inhibitors, and some DNMT inhibitors present low specificity and cannot directly block the active site of DNMT. Therefore, in this present study, we used genetic modulation to interfere with DNMT1 to explore its mechanism in NP.

Several signaling pathways, including the phosphatidylinositol 3-kinase/protein kinase B (PI3K/Akt) pathway, are critical for microglia activation and inflammation [29–31]. The activation of the PI3K/Akt pathway contributes to the release of pro-inflammatory cytokines (TNF-α, IL-1β, and IL-6), as well as iNOS expression [32]. The blocking of the PI3K/Akt pathway mitigates lipopolysaccharide (LPS)-induced neuroinflammation and reduces the levels of inflammatory factors such as TNF-α and IL-6 [33].

On a separate note, the PI3K/Akt pathway is pivotal for the development and maintenance of NP [34]. The blockade of this pathway exerts a mitigating effect on NP caused by surgery [35], suppresses inflammatory responses [36], and ameliorates cognitive impairment in NP rats [37]. Furthermore, the PI3K/Akt pathway is activated in CCI rat models [38], which participates in the activation of microglia [39] and M2 polarization of microglia [40, 41]. JTC-801 prominently impedes the Akt activation and diminishes the expressions of inflammatory factors, thereby palliating mechanical allodynia in paclitaxel-elicited NP [42]. Hence, suppression of the PI3K/Akt pathway probably is a primary therapeutic target for NP. Additionally, prior research has documented the promotion effect of DNMT1 on PI3K/Akt pathway activation [43]. Nevertheless, more studies remain to be carried out on whether suppression of DNMT1 by RNA interference represses NP via the modulation of microglia M2 polarization by blocking the PI3K/Akt pathway. Herein, this present study probed into the action of DNMT1 siRNA in NP by regulating microglia M2 polarization, expecting to offer potential intervention therapeutic targets for NP treatment.

## Materials and methods

### Ethics statement

All animal experiments were approved by the Ethics Committee for *Animal Care and Use* of Weifang Traditional Chinese Medicine Hospital. Significant efforts were taken to minimize the number of animals and their suffering. All methods were performed under the relevant guidelines and ARRIVE guidelines.

### Experimental animals

Specific pathogen-free Sprague–Dawley healthy male rats (8 weeks old, weighing 200–220 g) were provided by Charles River (License No. SCXK (Beijing) 2021–0006, Beijing, China). Rats were reared in a temperature-controlled (20–25 °C) environment under 12 h light–dark cycles, with free access to food and water.

### Establishment of NP models

NP rat models were constructed through chronic constriction injury (CCI) [44]. Rats were firstly anesthetized with an intraperitoneal injection of 50 mg/kg of 2% sodium pentobarbital (Sigma-Aldrich, Merck KGaA, Darmstadt, Germany). Next, the sciatic nerve was exposed by blunt dissection [45], and then 4 loose knots were made around the nerve at a 1-mm interval from the proximal end of the spine to the bifurcation using the 4–0 chromic catgut. The ligatures were tightened until the presence of slight fibrillation in the operated limb, followed by suture using 4–0 sterile surgical sutures. The sciatic nerve of rats in the sham group was exposed for the same period, but not ligated.

### Animal treatment and grouping

Total 32 rats were arbitrarily allocated into 4 groups (8 rats/group): sham group, CCI group, CCI + siRNA-Ctrl group, and CCI + siRNA-DNMT1 group. Previous studies have revealed that the lateral ventricular delivery of siRNA effectively silenced the expression of target genes in the brain, with a range of 50–80% [46, 47]. Before CCI modeling, the rat head was fixed with a stereotaxic instrument and a small 1-mm-diameter hole was drilled on the right side of the rat skull; next, the lateral ventricle of rats was injected with 2 μL normal saline containing 20 nM siRNA-Ctrl or DNMT1 siRNA at a rate of 0.5 μL/min and then the needle stayed for 5 min in the brain after injection to prevent leakage; finally, bone wax was used to seal the burr hole and the incision was sutured [14]. The sham and CCI groups received an equivalent amount of normal saline. On the 14^th^ day after CCI induction, rats were euthanized by injecting pentobarbital sodium (50 mg/kg) under anesthesia, and L4–L6 spinal cord samples were isolated for subsequent analyses [12]. Thereafter, 4 rats were used for immunofluorescence and another 4 rats were used for tissue homogenate, followed by reverse transcription quantitative polymerase chain reaction (RT-qPCR), Western blot, and enzyme-linked immunosorbent assay (ELISA). All the above procedures were performed under deep anesthesia. Additionally, the construction and packaging of DNMT1 siRNA and its negative control (NC) siRNA-Ctrl were performed by GenePharma (Shanghai, China).

### Determination of pain threshold

All rats were habituated to the environment prior to behavioral testing [44]. The mechanical withdrawal threshold (MWT) and thermal withdrawal latency (TWL) of rats were measured on days 0, 3, 7, and 14 after CCI induction [9]. The 0-day was considered the day before CCI induction. A series of Von Frey filaments (2, 4, 6, 8, 10, and 15 g) (North Coast Medical, San Jose, CA, USA) was utilized to determine MWT value [16]. A plantar algometer (Tes7370, Ugo Basile, Comerio, Italy) was used to measure TWL value on the plantar surface of rat paws.

### Immunofluorescence staining

Rat L4-L6 spinal cord samples were fixed, dehydrated, and frozen in a -80 °C freezer, and cryosectioned at 8 μm thickness for subsequent studies. The removed sections were dried for 10 min and rinsed thrice with phosphate-buffered saline (PBS) for 10 min each. Sections were rinsed thrice for 10 min each after breaking the membranes with 0.5% Triton for 2 h. Following repair using citrate solution, sections were naturally cooled and then rinsed thrice for 10 min each. Subsequently, sections was blocked with 2% bovine serum albumin and incubated overnight with following primary antibodies: anti-ionized calcium-binding adaptor molecule-1 (Iba-1) (red; 1:100, ab283319, Abcam, Cambridge, UK), anti-DNMT1 (green; 1:250, ab188453, Abcam), anti-iNOS (green; 1:250, ab178945, ab283319, Abcam), anti-Arg-1 (green; 1:200, ab96183, Abcam) at 4 °C, followed by washing with 0.01 moL/L PBS. Afterwards, sections were reacted for 1 h with secondary antibody immunoglobulin G (IgG, 1:2000, ab205718, Abcam) at 37 °C under dark conditions, and then washed. Following staining the nuclei with Hoechst for 10 min, sections were rinsed five times for 10 min each. The expression and localization of DNMT1, iNOS, and Arg-1 in microglia were determined after mounting using glycerol, with Iba-1 as a marker of microglia.

### Cell culture and transfection

Rat microglial cell line (highly aggressive proliferating immortalized, HAPI) provided by BeNa Culture Collection (Xinyang, Henan, China) was cultured in high-glucose Dulbecco’s modified Eagle’s medium containing 10% fetal bovine serum (Thermo Fisher Scientific, Waltham, MA, USA) in an incubator with 5% CO_2_ and 95% humidity.

Cells were allocated into the following 7 groups: control group, LPS group (treated with 100 ng/mL LPS for 24 h), LPS + si-NC group (transfected with si-NC for 24 h, followed by LPS treatment for 24 h), LPS + si-DNMT1 group (transfected with si-DNMT1 for 24 h, followed by LPS treatment for 24 h), LPS + oe-NC group (transfected with oe-NC for 24 h, followed by LPS treatment for 24 h), LPS + oe-DNMT1 group (transfected with oe-DNMT1 for 24 h, followed by LPS treatment for 24 h), and LPS + si-DNMT1 + IGF-1 group (based on the LPS + si-DNMT1 group, cells were treated with Akt activator (insulin-like growth factor 1, IGF-1, 100 ng/mL) for 1 h [48]. IGF-1 (Enzyme-linked Biotechnology, Shanghai, China), si-DNMT1, si-NC, oe-DNMT1, and oe-NC (all from GenePharma) were transfected into HAPI cells using Lipofectamine 2000 (Invitrogen, Carlsbad, CA, USA) at 50 nM.

### ELISA

Rat L4-L6 spinal cord samples or HAPI cells were lysed using radio-immunoprecipitation assay (RIPA) lysis buffer containing protease inhibitors (Solarbio, Beijing, China) and centrifuged (14,000 × g, 5 min) to obtain the supernatant. Subsequently, the levels of inflammatory factors TNF-α (ml002953), IL-1β (ml037361), IL-6 (ml064292), IL-10 (ml028497) in tissue homogenate or cell supernatant were measured using ELISA kits (Enzyme-linked Biotechnology).

### RT-qPCR

Total RNA in L4-L6 spinal cord samples and HAPI cells was extracted using the TRIzol method (Invitrogen). The RNA concentration was detected utilizing NanoDrop spectrophotometer (Thermo Fisher Scientific) and total RNA was reverse-transcribed to cDNA using PrimeScript RT reagent kits (Takara, Dalian, Liaoning, China). PCR amplification reactions were conducted using Dy NAmo™ SYBR® Green qPCR kits (Finnzymes, Espoo, Finland) on a Bio-Rad CFX96 fluorescence qPCR instrument (ABI, Carlsbad, CA, USA). The reaction conditions were as follows: pre-denaturation at 95 °C for 10 min, and then 40 cycles of denaturation at 95 °C for 10 s, annealing at 60 °C for 20 s, and extension at 72 °C for 34 s. Glyceraldehyde-3-phosphate dehydrogenase (GAPDH) acted as the internal control and the data were analyzed using the 2^−ΔΔCt^ method. The primer sequences are detailed in Table 1.

**Table 1:** Primer sequences of RT-qPCR

Gene	Forward 5’-3’	Reverse 5’-3’
DNMT1	GAGCTACCACGCAGACATCA	CGAGGAAGTAGAAGCGGTTG
iNOS	CGAAACGCTTCACTTCCAA	TGAGCCTATATTGCTGTGGCT
Arg-1	AACACGGCAGTGGCTTTAACC	GGTTTTCATGTGGCGCATTC
GAPDH	GGTCTCCTCTGACTTCAACA	GTGAGGGTCTCTCTCTTCCT

*RT-qPCR* Reverse transcription quantitative polymerase chain reaction, *DNMT1* DNA methyltransferase 1, *iNOS* Inducible nitric oxide synthase, *Arg-1* Arginase-1, *GAPDH* Glyceraldehyde-3-phosphate dehydrogenase

### Flow cytometry

The percentage of M1/M2 microglia phenotype was determined by evaluating the levels of their corresponding markers CD16 and CD206. Firstly, rat L4-L6 spinal cord samples were cut with scissors, detached with 0.15% trypsin, and repeatedly blown into single-cell suspension, followed by isolation of mononuclear cells by Percoll density gradient centrifugation. The mononuclear cells were then resuspended in PBS and stained with fluorescence-labeled CD16 (ab246222, Abcam) and CD206 antibodies (ab270647, Abcam) for 30 min at 4 °C in the dark. For cell culture samples, HAPI cells were collected, rinsed with PBS, adjusted to 1 × 10^6^ cells/mL, and stained with the same CD16 (Abcam) and CD206 (Abcam) antibodies. Later, the percentages of CD16 and CD206 were measured by a FACSCalibur flow cytometer equipped with FlowJo software (version vX 0.7) (Beckman Coulter, Brea, CA, USA).

### Western blot

RIPA lysis buffer (Beyotime, Shanghai, China) was utilized to lyse rat L4-L6 spinal cord samples or HAPI cells, and then the protein concentration was measured with bicinchoninic acid protein assay kits (Beyotime). Proteins were separated using 10% sodium dodecyl sulfate–polyacrylamide gel electrophoresis and subsequently electrotransferred to polyvinylidene fluoride (PVDF) membranes (Millipore, Bedford, MA, USA). Afterwards, 5% skim milk was configured using Tris-buffered saline Tween-20 (TBST), and then the PVDF membranes were put into milk, followed by shaking and blocking for 1 h at room temperature to block non-specific binding. Thereafter, membranes were incubated overnight at 4 °C with primary antibodies: anti-DNMT1 (1:1000, ab188453, Abcam), anti-PI3K (1:1000, ab32089, Abcam), anti-p-PI3K (1:1000, ab278545, Abcam), anti-Akt (1:500, ab8805, Abcam), and anti-p-Akt (1:1000, ab38449, Abcam). Following washing twice with TBST, membranes were reacted for 1 h with horseradish peroxidase-labeled goat anti-rabbit secondary antibody IgG (1:2000, ab48386, Abcam) at room temperature. They were then developed with enhanced chemiluminescence working solution (Millipore) and photographed. The density of protein bands was detected with Image J software (version 1.48, NIH, Bethesda, MD, USA), with β-actin as the internal reference.

### Statistical analysis

All data were processed using SPSS 21.0 statistical software (IBM Corp, Armonk, NY, USA). Measurement data were represented as mean ± standard deviation (SD). Data between two groups were analyzed using the independent sample *t*-test or one-way repeated measures analysis of variance (ANOVA), with one-way ANOVA for comparisons among multiple groups. Tukey’s multiple comparisons test was conducted for the post hoc analysis. The *p* < 0.05 indicated statistical significance.

## Results

### DNMT1 was upregulated in microglia of CCI rats

**Fig. 1:**
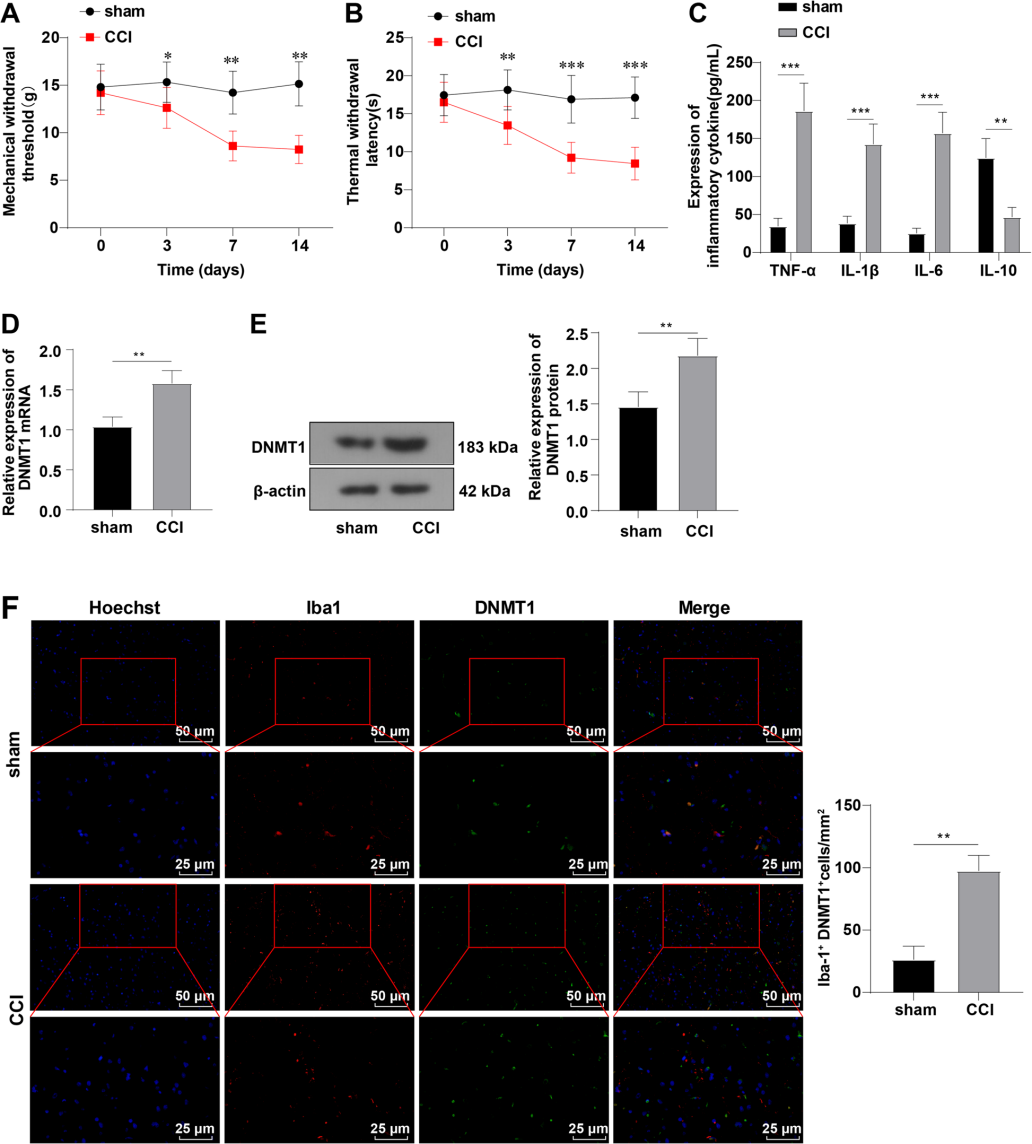
DNMT1 was upregulated in microglia of CCI rats. Rat models of NP were established by CCI. **A-B** MWT and TWL values measured on days 0, 3, 7, and 14; **C** Levels of inflammatory factors TNF-α, IL-1β, IL-6, and IL-10 measured by ELISA; **D** Expression of DNMT1 mRNA determined by RT-qPCR; **E** Protein level of DNMT1 determined by Western blot; **F** Expression and localization of DNMT1 in microglia determined by immunofluorescence (Red, Iba-1; green, DNMT1). *N* = 4, data were presented as mean ± SD. One-way repeated measures ANOVA was used to analyze the data in panels A/B and the *t*-test was used to analyze the data in panels C/D/E//F. *p < 0.05, **p < 0.01, ***p < 0.001

Compelling evidence has revealed the regulation of DNMT1 in NP onset [21]. To estimate the role of DNMT1 in NP, we established rat models of NP by CCI and firstly assessed the pain threshold of CCI rats by measuring MWT and TWL. The results revealed that CCI rats presented lower values of MWT and TWL than the sham group (all *p* < 0.01) (Fig. 1A-B). Additionally, to further assess the neuroinflammation in CCI rats, we measured the levels of TNF-α, IL-1β, IL-6, and IL-10 using ELISA, which demonstrated that the CCI group exhibited prominently higher contents of pro-inflammatory cytokines TNF-α, IL-1β, and IL-6 and lower level of anti-inflammatory cytokine IL-10 than the sham group (all *p* < 0.01) (Fig. 1C). Next, we measured DNMT1 expression levels and noted remarkably upregulated DNMT1 mRNA and protein levels in the CCI group compared with the sham group (*p* < 0.01) (Fig. 1D-E). Thereafter, immunofluorescence was employed to detect the localization of DNMT1 in microglia, which revealed noticeably highly-expressed DNMT1 in the plasma membrane of activated microglia (*p* < 0.01) (Fig. 1F). Overall, DNMT1 was upregulated in the microglia of CCI rats.

### DNMT1 siRNA alleviated CCI-induced NP

**Fig. 2:**
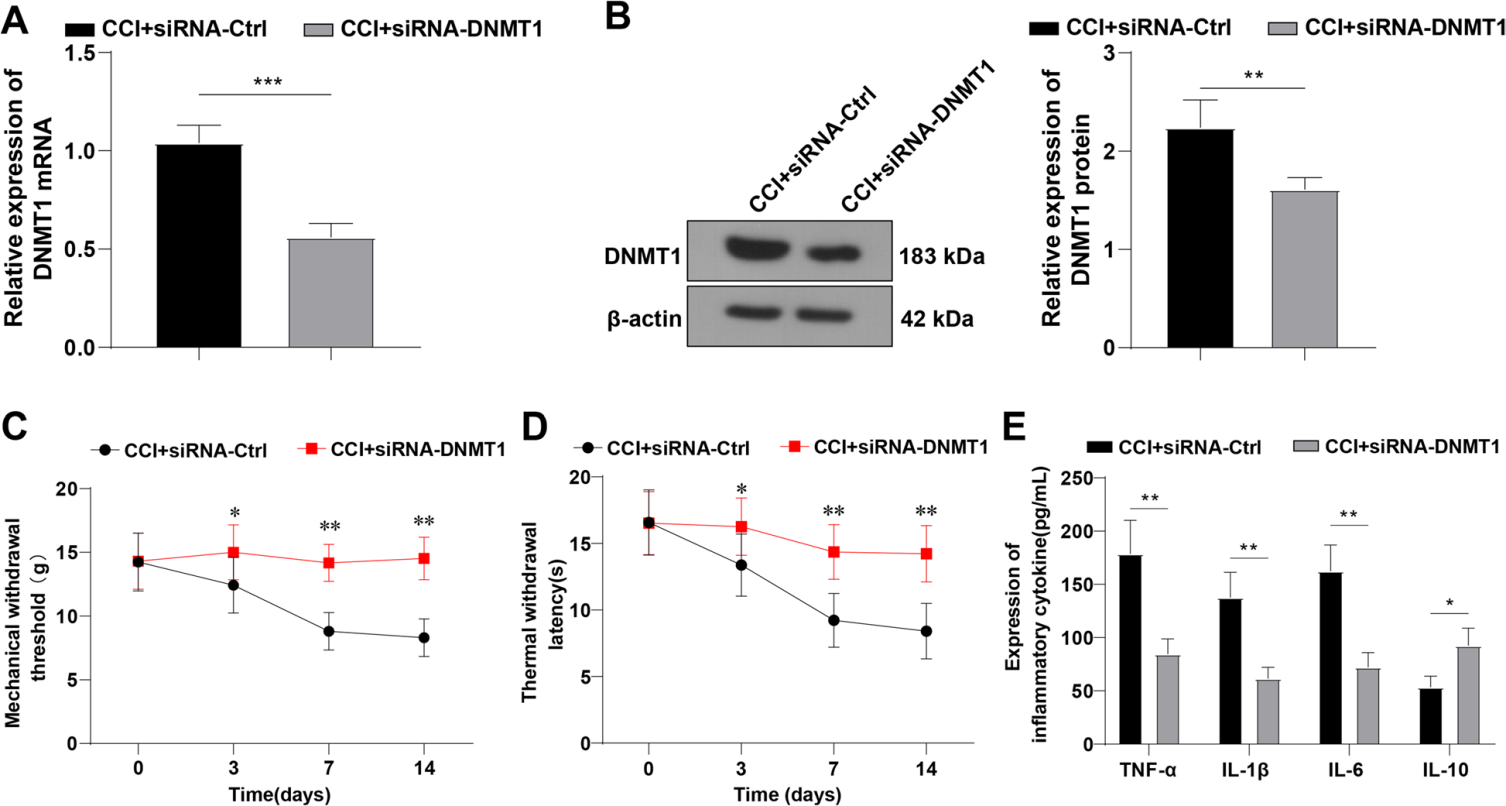
DNMT1 siRNA alleviated CCI-induced NP. CCI rats were treated with DNMT1 siRNA. **A** Expression of DNMT1 mRNA determined by RT-qPCR; **B** Protein level of DNMT1 determined by Western blot; **C-D** Measurement of MWT and TWL; **E** Levels of TNF-α, IL-1β, IL-6, and IL-10 determined using ELISA. *N* = 4, data were expressed as mean ± SD. The *t*-test was adopted to analyze the data in panels A/B/E and one-way repeated measures ANOVA was adopted to analyze the data in panels C/D. *p < 0.05, **p < 0.01, ***p < 0.001

With the purpose to investigate the action of DNMT1 in NP, CCI rats were injected with 2 μL normal saline containing 20 nM DNMT1 siRNA to interfere with DNMT1 expression. Firstly, RT-qPCR and Western blot assays unveiled that DNMT1 mRNA and protein levels were significantly reduced in the CCI + siRNA-DNMT1 group compared with the CCI + siRNA-Ctrl group (*p* < 0.01) (Fig. 2A-B), indicating the successful interference of DNMT1 expression. Subsequently, we observed that the CCI + siRNA-DNMT1 group had higher MWT and TWL than the CCI + siRNA-Ctrl group (all *p* < 0.01) (Fig. 2C-D). Finally, ELISA detection unraveled that the CCI + siRNA-DNMT1 group exhibited lower TNF-α, IL-1β, and IL-6 contents and higher IL-10 levels than the CCI + siRNA-Ctrl group (all *p* < 0.01) (Fig. 2E). The aforementioned results indicated the mitigating effect of RNA interference-mediated DNMT1 knockdown on CCI-induced NP.

### DNMT1 siRNA promoted microglia M2 polarization in CCI rats

**Fig. 3:**
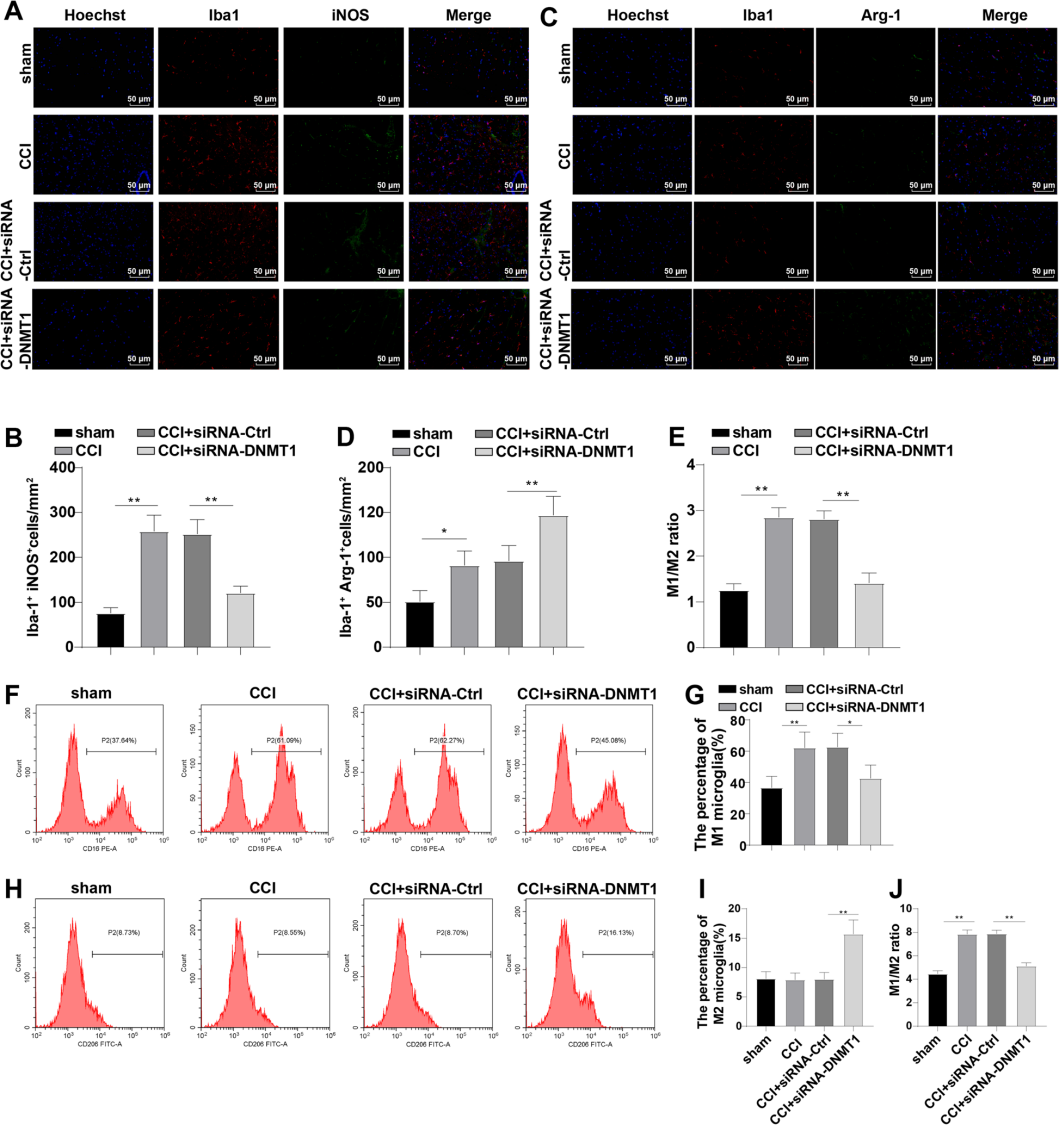
DNMT1 siRNA promoted microglia M2 polarization in CCI rats. **A-D** Expression and localization of iNOS and Arg-1 in microglia detected by immunofluorescence (red, Iba-1; green, iNOS and Arg-1); **E** Ratio of M1/M2 microglia; **F-I** Percentages of M1 microglia phenotype (CD16) and M2 microglia phenotype (CD206) detected using flow cytometry; **J** Ratio of M1/M2 microglia. *N* = 4, data were exhibited as mean ± SD. Data among multiple groups were analyzed by one-way ANOVA, followed by Tukey’s test. *p < 0.05, **p < 0.01, ***p < 0.001

Subsequently, we detected the levels of M1 marker (iNOS) and M2 marker (Arg-1) in rats to investigate the effect of CCI induction and DNMT1 siRNA on M2 polarization of rat microglia by immunofluorescence. The results demonstrated that compared with the sham group, the CCI group had increased Iba-1^+^iNOS^+^ cells (*p* < 0.01), slightly elevated Iba-1^+^ Arg-1^+^ cells (*p* < 0.05), and clearly raised M1/M2 ratio (*p* < 0.01), while DNMT1 siRNA contributed to significantly decreased number of Iba-1^+^iNOS^+^ cells, increased Iba-1^+^ Arg-1^+^ cells, and diminished M1/M2 ratio (all *p* < 0.01) (Fig. 3A-E). In addition, we examined M1 microglial phenotype (CD16) and M2 microglia phenotype (CD206) using flow cytometry and observed that CCI induction significantly increased the proportion of M1 microglia (*p* < 0.01) but had no obvious effect on the percentage of M2 microglia (*p* > 0.05), and DNMT1 siRNA significantly diminished the proportion of M1 microglia and increased the percentage of M2 microglia, resulting in the decreased ratio of M1/M2 (all *p* < 0.01) (Fig. 3F-J). Altogether, CCI induction resulted in M1 polarization of rat microglia, while inhibition of DNMT1 facilitated M2 polarization of CCI rat microglia.

### The effects of DNMT1 knockdown and overexpression on M1/M2 polarization in LPS-induced HAPI microglia

**Fig. 4:**
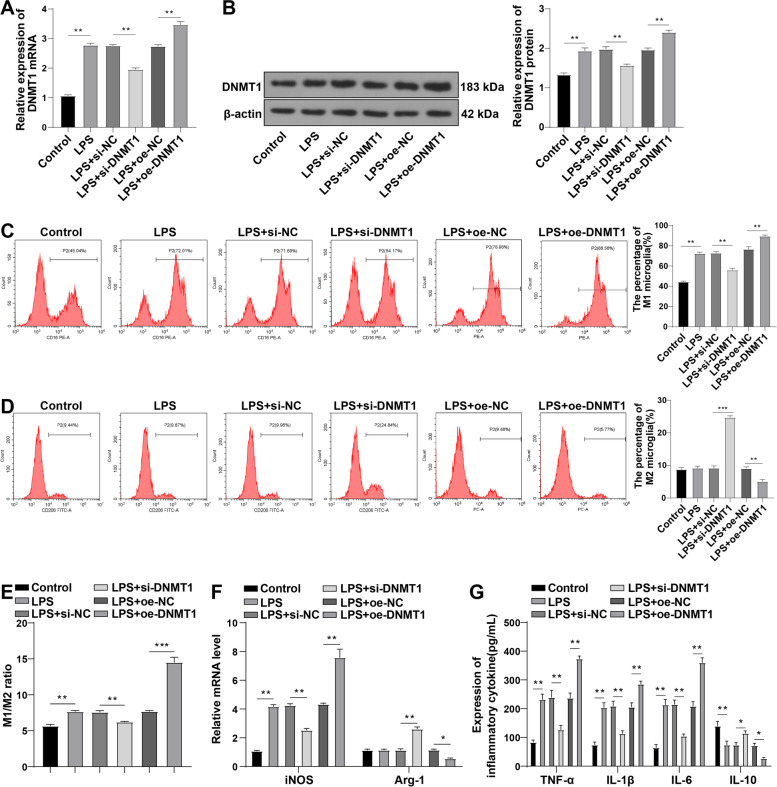
DNMT1 siRNA prevented LPS-induced M1 polarization and accelerated M2 polarization in HAPI cells. **A** RT-qPCR determined the expression of DNMT1 mRNA in HAPI cells; **B** Western blot determined the protein level of DNMT1; **C-D** Flow cytometry detected the percentages of M1 microglia phenotype (CD16) and M2 microglia phenotype (CD206); **E** Ratio of M1/M2 microglia; **F** RT-qPCR measured the relative mRNA levels of iNOS and Arg-1; **G** ELISA determined the levels of TNF-α, IL-1β, IL-6, and IL-10. Cell experiment was conducted 3 times; data were shown as mean ± SD. Data among multiple groups were analyzed by one-way ANOVA, followed by Tukey’s test. *p < 0.05, **p < 0.01, ***p < 0.001

To further elucidate the specific role of DNMT1 in regulating M1/M2 polarization of microglia, the LPS-induced HAPI cells were transfected with si-DNMT1 or oe-DNMT1. Firstly, RT-qPCR and Western blot showed that compared with the control group, DNMT1 mRNA and protein levels were remarkably elevated in the LPS group; after interfering with DNMT1 expression in HAPI cells, DNMT1 levels were prominently reduced, while the LPS + oe-DNMT1 group showed notably higher DNMT1 levels than the LPS oe-NC group (all *p* < 0.01) (Fig. 4A-B). Flow cytometry revealed that LPS treatment significantly increased the percentage of M1 microglia (CD16) and the ratio of M1/M2 microglia, and DNMT1 siRNA prominently exerted contrary effects (all *p* < 0.01); additionally, the percentage of M2 microglia showed no significant change upon LPS treatment, but increased in the LPS + si-DNMT1 group relative to the LPS + si-NC group (*p* < 0.01) (Fig. 4C-E). Moreover, RT-qPCR and ELISA illustrated that LPS resulted in significant increases in M1 markers (iNOS, TNF-α, IL-1β, and IL-6), indicating that LPS promoted microglia polarization to the M1 phenotype. After silencing of DNMT1, iNOS, TNF-α, and IL-6 levels were markedly decreased, while M2 markers (Arg-1 and IL-10) were increased (all *p* < 0.01) (Fig. 4F-G). In contrast, overexpression of DNMT1 exerted the opposite results (Fig. 4A-G). Briefly, DNMT1 interference facilitated the M1-to-M2 polarization of microglia, whereas DNMT1 overexpression suppressed this M1-to-M2 polarization.

### DNMT1 siRNA promoted microglia M2 polarization by blocking the PI3K/Akt pathway

**Fig. 5:**
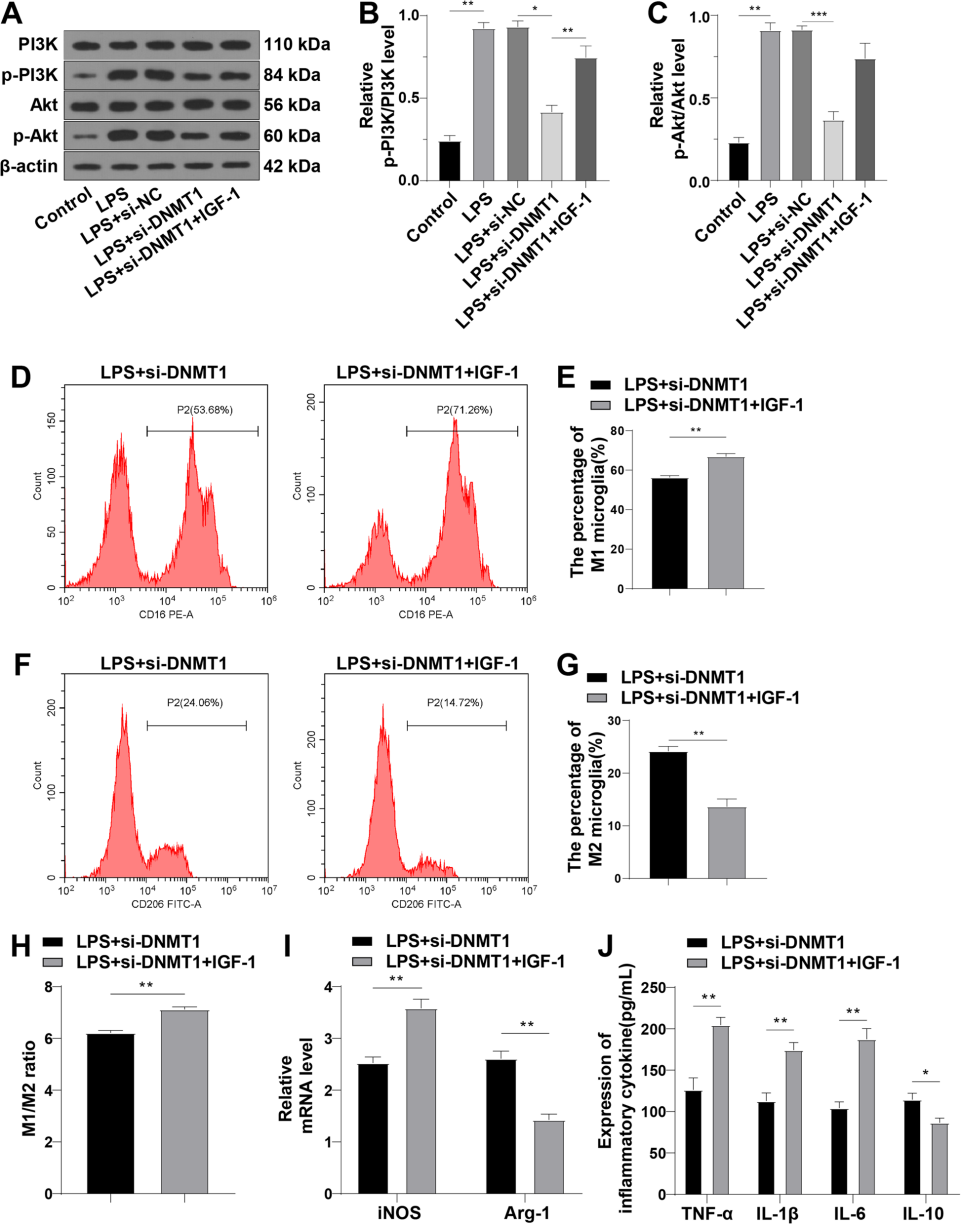
DNMT1 siRNA promoted microglia M2 polarization by blocking the PI3K/Akt pathway. **A-C** Protein levels of PI3K, p-PI3K, Akt, and p-Akt measured by Western blot. **D-G** M1 microglia phenotype (CD16) and M2 microglia phenotype (CD206) detected using flow cytometry; **H** Ratio of M1/M2 microglia; **I** The relative mRNA levels of iNOS and Arg-1 measured by RT-qPCR; **J** Levels of TNF-α, IL-1β, IL-6, and IL-10 determined by ELISA. Cell experiment was conducted 3 times; data were exhibited as mean ± SD. Data among multiple groups were analyzed by one-way ANOVA, followed by Tukey’s test. *p < 0.05, **p < 0.01, ***p < 0.001

To explore the downstream mechanism of DNMT1 siRNA in regulating M2 polarization of microglia, we treated LPS-induced HAPI cells with DNMT1 siRNA and Akt activator IGF-1. Western blot analysis unveiled that the LPS group had higher levels of p-PI3K/PI3K and p-Akt/Akt than the control group, DNMT1 siRNA significantly decreased p-PI3K/PI3K and p-Akt/Akt levels, while the LPS + si-DNMT1 + IGF-1 group exhibited higher p-PI3K/PI3K and p-Akt/Akt than the LPS + si-DNMT1 group (all *p* < 0.01) (Fig. 5A-C), indicating that DNMT1 siRNA inhibited LPS-stimulated PI3K/Akt pathway activation and IGF-1 activated the PI3K/Akt pathway. Subsequently, we explored the effect of the PI3K/Akt pathway on M2 polarization. Compared with the LPS + si-DNMT1 group, the LPS + si-DNMT1 IGF-1 group had a markedly increased percentage of M1 microglia, M1/M2 ratio, and M1 markers (iNOS, TNF-α, IL-1β, and IL-6), but diminished M2 microglia and M2 markers (Arg-1 and IL-10) (all *p* < 0.01) (Fig. 5D-J). In summary, DNMT1 siRNA facilitated microglia M2 polarization by impeding the PI3K/Akt pathway.

## Discussion

NP is a frequent clinical chronic pain, which also remains a refractory disease that causes a considerable burden on the somatic and mental health of patients [49, 50]. Transfer of microglia polarization from M1 to M2 phenotype is considered a prospective therapeutic strategy for NP [51]. Additionally, DNA methylation affects the development of NP and DNMT1 is a principle isoform of DNMTs to sustain DNA methylation in mammals [44]. The PI3K/Akt pathway is necessitated for NP progression and maintenance [52]. This study highlighted the function of DNMT1 silencing in NP via regulating microglia M2 polarization and PI3K/Akt pathway.

MWT and TWL are widely recorded to assess the mechanical hypersensitivity and thermal hyperalgesia [53], and lower MWT and TWL are indicative of successful CCI models [54]. Firstly, we successfully established NP rat models via CCI, as manifested by reduced MWT and TWL values. NP is commonly featured by neuroinflammation, and its pathogenesis involves the increased levels of pro-inflammatory cytokines (including TNF-α, IL-6, COX-2, and IL-1β) and decreased anti-inflammatory cytokines (such as IL-4 and IL-10) [55]. Hence, we detected the inflammation and found elevated TNF-α, IL-1β, and IL-6 levels, and decreased IL-10 in CCI rats as expected. TNF-α is imperative in regulating immediate and ongoing stages of NP and the administration of TNF-α or IL-6 evokes thermal hyperalgesia and mechanical allodynia in rats [56]. Due to the substantial involvement of DNMT1 in NP as existing research reported [57], we measured the expression of DNMT1 and noted an upregulated DNMT1 in activated microglia of CCI rats, which was similar to the findings of Sarah L et al. [23]. Furthermore, we administrated CCI rats with DNMT1 siRNA to explore the function of DNMT1 in NP. The results unveiled that DNMT1 siRNA enhanced MWT and TWL values and suppressed neuroinflammation in CCI rats. Preceding evidence has elicited that DNA methylation regulates inflammation by epigenetically mediating signal transduction molecules, including myeloid differentiation factor 88 (MyD88), tumor necrosis factor receptor-associated factor 6 (TRAF6), and Toll-like receptor 2 (TLR2) [58]. Interestingly, blockade of DNA methylation predominantly impacts pain behaviors during NP and inflammatory pain, and additionally, DNMT inhibitor reduces incision-induced mechanical hypersensitivity and thermal sensitivity and alleviates hind paw swelling and inflammation [59]. Meanwhile, DNMT1 in the injured dorsal root ganglia may be warranted for NP genesis and genetic knockdown or pharmacological suppression of DNMT1 extenuates nerve injury-elicited pain hypersensitivities; for instance, injection of RG108, a DNMT1 inhibitor, palliates spinal nerve ligation-evoked heat hyperalgesia, cold allodynia, and mechanical allodynia [21]. Taken together, DNMT1 siRNA could attenuate CCI-elicited NP.

Microglia activation and following inflammatory responses are paramount in NP advance and the transformation of microglia polarization to pro-inflammatory phenotype normally presents during neuroinflammation [60]. M1 polarization markers include iNOS and CD16, and M2 markers consist of Arg-1 and CD206 [61]. Hence, we detected the microglia M1 and M2 polarization. In CCI rats, we observed notably increased iNOS and M1 microglia, slightly raised Arg-1, but an elevated M1/M2 ratio. However, DNMT1 siRNA prominently diminished iNOS and M1 microglia, elevated Arg-1 and M2 microglia, and decreased M1/M2 ratio. Generally, following peripheral nerve damage, microglia in the adult spinal cord inclines to the M1 phenotype and over-generates pro-inflammatory mediators (such as IL-1β and TNF-α), which greatly sensitizes the spinal cord neurons and produces pain, and consequently, shifting from M1 to the M2 polarization of spinal cord microglia is effective in ameliorating NP [62]. Depletion of DNMT1 can alleviate AD-associated NP by inducing M2 polarization of microglia [15]. Briefly, CCI induction contributed to the M1 polarization of microglia, and DNMT1 siRNA promoted M2 polarization in CCI rats. We further elucidated the role of DNMT1 via in vitro experiments. HAPI cells were treated with LPS and transfected with si-DNMT1 or oe-DNMT1. The results were consistent with those in vivo studies. DNMT1 interference augmented the M1-to-M2 polarization of microglia, whereas DNMT1 overexpression exerted the opposite effects.

Preceding research has reported that the PI3K/Akt pathway, one of the most important microglial intracellular cascades, is implicated in NP [34] and microglia M2 polarization [40, 41]. Subsequently, we explored the downstream mechanism of DNMT1 siRNA in microglia M2 polarization. Based on our results, the phosphorylation of the PI3K/Akt pathway-related proteins was raised in LPS-treated HAPI cells but diminished upon treatment of DNMT1 siRNA. Furthermore, we noted that IGF-1 (the Akt activator) elevated M1 microglia and M1/M2 ratio and decreased M2 microglia. Consistently, CCI contributes to an increase in levels of p-PI3K and p-Akt, and inhibiting the PI3K/Akt pathway exerts anti-inflammatory responses, ultimately suppressing NP [36]. Furthermore, the PI3K/Akt pathway effectively regulates microglia polarization and LY294002, an Akt inhibitor, enhances M1 marker expression [63]. Previous research has demonstrated that DNMT1 could activate the PI3K/Akt/mammalian target of rapamycin (mTOR) pathway in diabetic retinopathy [64])and 3,6-dihydroxyflavone (3,6-DHF), an effective DNMT1 inhibitor, suppresses the PI3K/Akt/mTOR pathway in breast carcinogenesis [65]. Collectively, DNMT1 siRNA facilitated microglia M2 polarization by blocking the PI3K/Akt pathway.

In conclusion, this study demonstrated that RNA interference-mediated DNMT1 silencing mitigated NP via the promotion of microglia M2 polarization by impeding the PI3K/Akt pathway. However, this paper only preliminarily elicited the function of DNMT1 knockdown on the PI3K/Akt pathway, lacking in-depth studies. Additionally, we merely detected the markers of microglia M1 and M2 polarization at one time point. Future studies shall be conducted to figure out the action of DNMT1 knockdown in modulating M2 polarization of microglia at the molecular level, such as the downstream microRNAs of DNMT1. Meanwhile, changes in microglia M1 and M2 polarization markers at different time points can be detected.

## Supplementary Information

**Additional file 1.**

**Additional file 2.**

**Additional file 3.**

**Additional file 4.**

**Additional file 5.**

**Additional file 6.**

**Additional file 7.**

**Additional file 8.**

**Additional file 9.**

**Additional file 10.**

**Additional file 11.**

**Additional file 12.**

## Data Availability

All data generated or analysed during this study are included in this published article.
