# Supplementary figures and images for: RNA interference-mediated silencing of DNA methyltransferase 1 attenuates neuropathic pain by accelerating microglia M2 polarization
Source: BMC Neurol. 2022 Oct 1;22:376. doi: 10.1186/s12883-022-02860-6 (PMC9526327; doi:10.1186/s12883-022-02860-6)

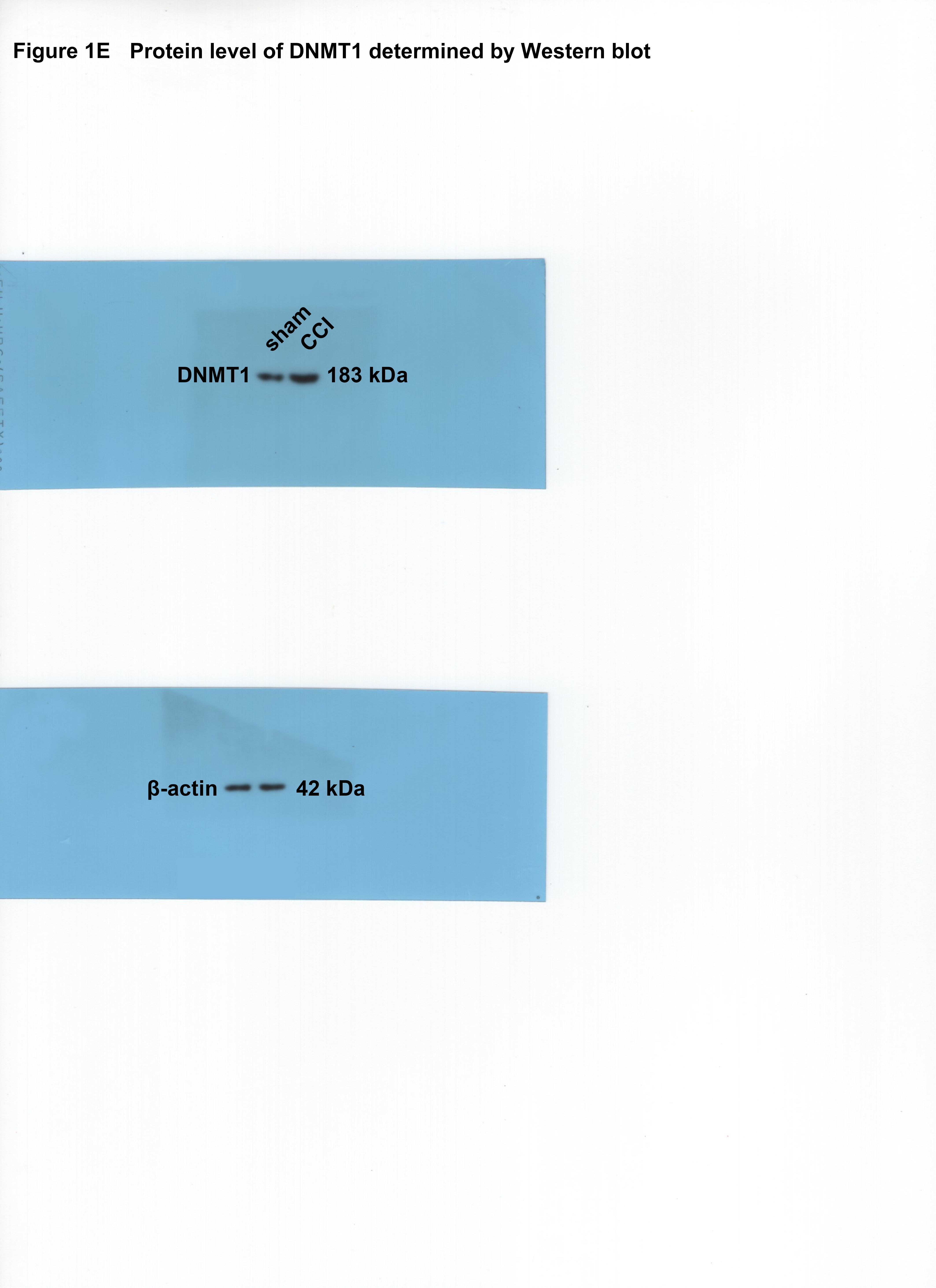

Supplement: Supplementary file 1 — Additional file 1. [file 12883_2022_2860_MOESM1_ESM.tif]

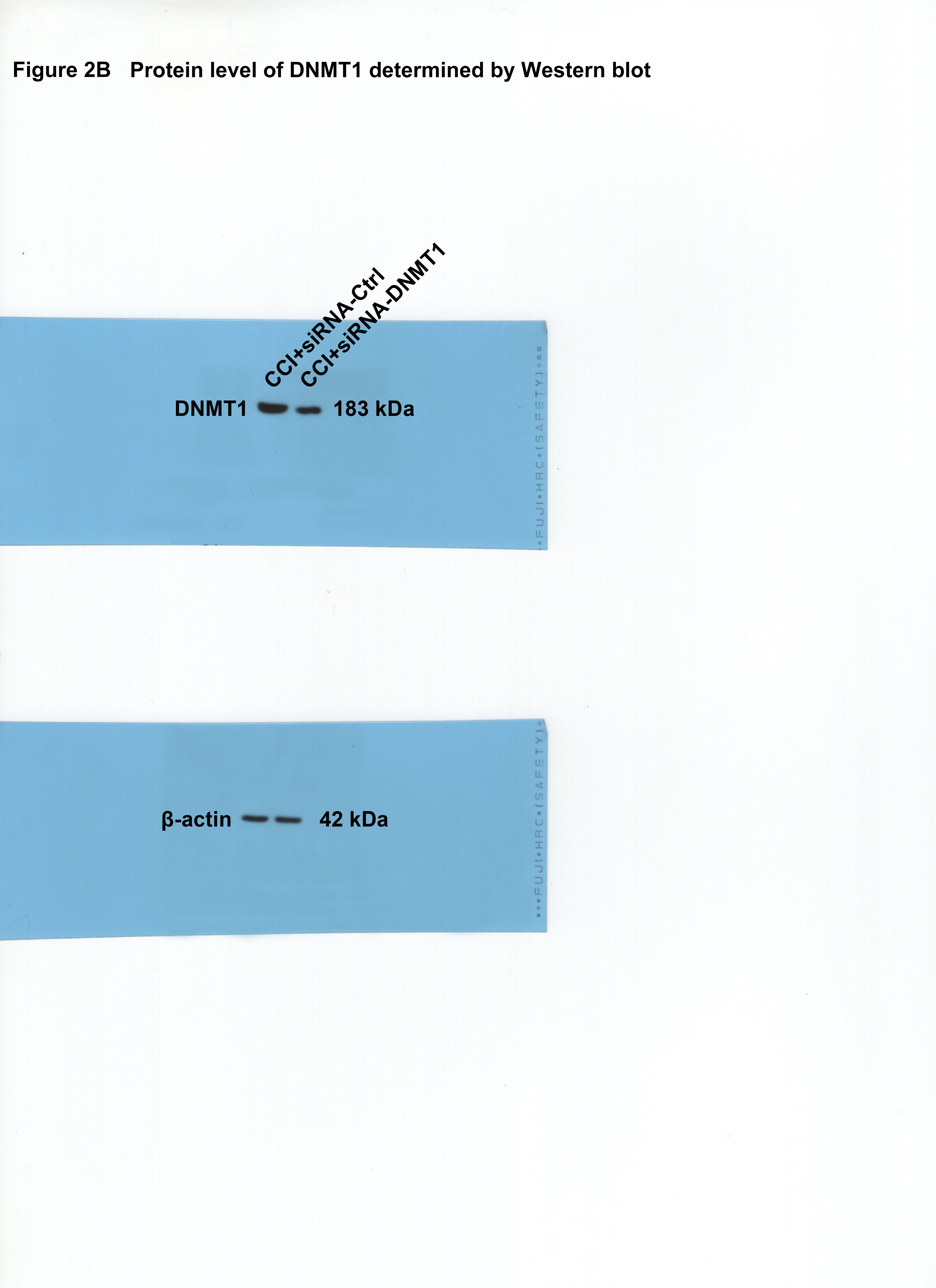

Supplement: Supplementary file 2 — Additional file 2. [file 12883_2022_2860_MOESM2_ESM.tif]

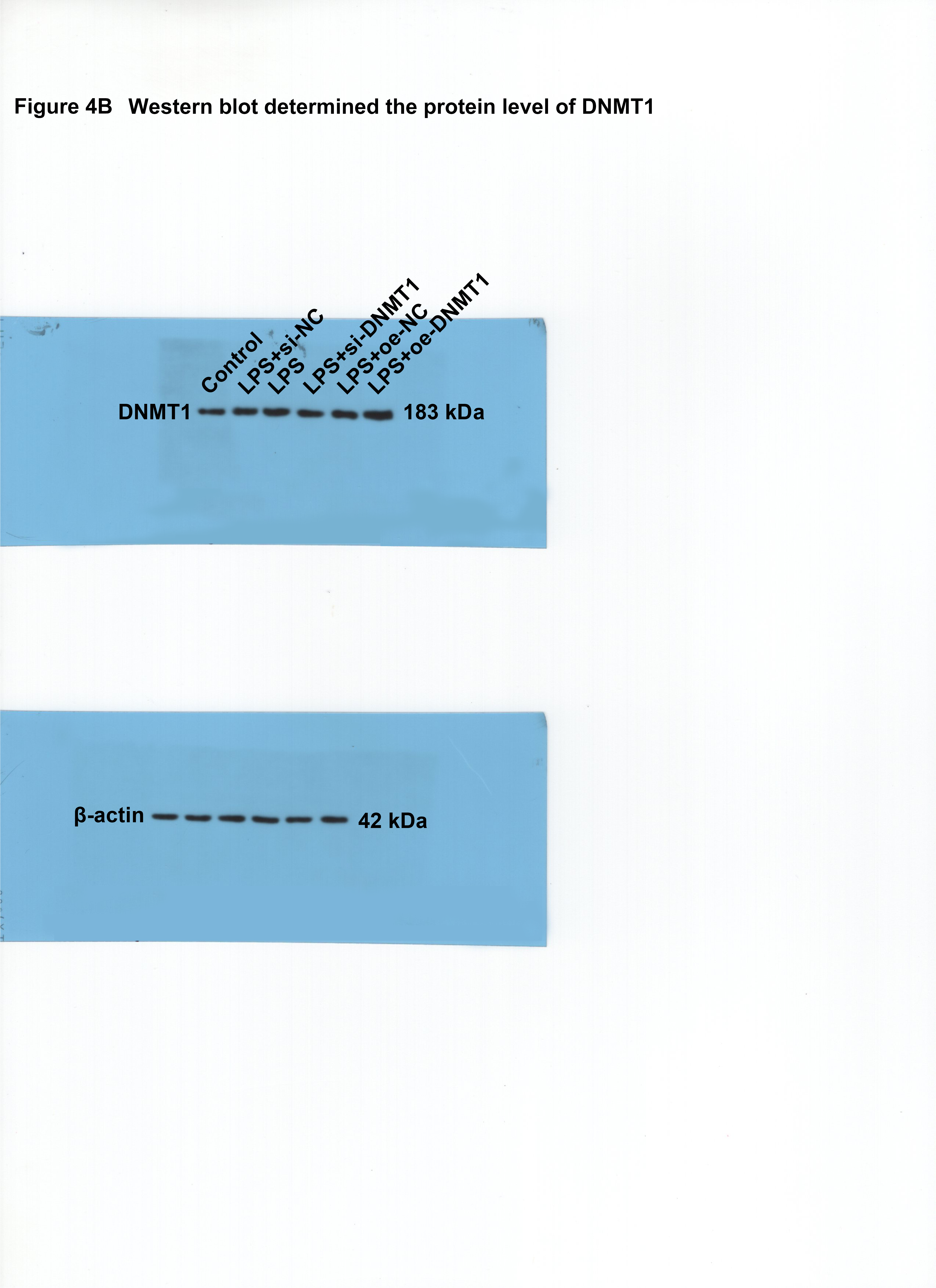

Supplement: Supplementary file 3 — Additional file 3. [file 12883_2022_2860_MOESM3_ESM.tif]

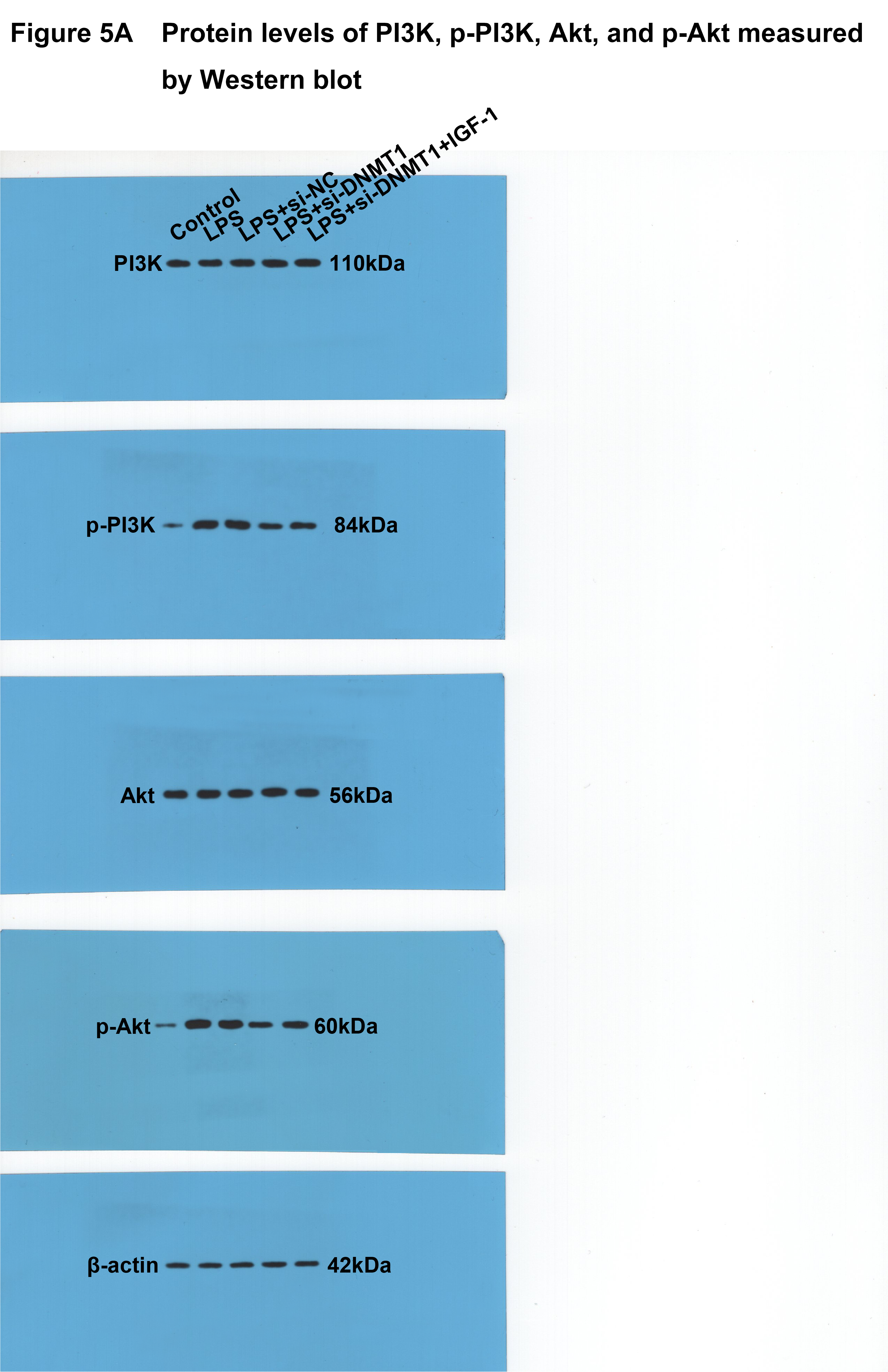

Supplement: Supplementary file 4 — Additional file 4. [file 12883_2022_2860_MOESM4_ESM.tif]

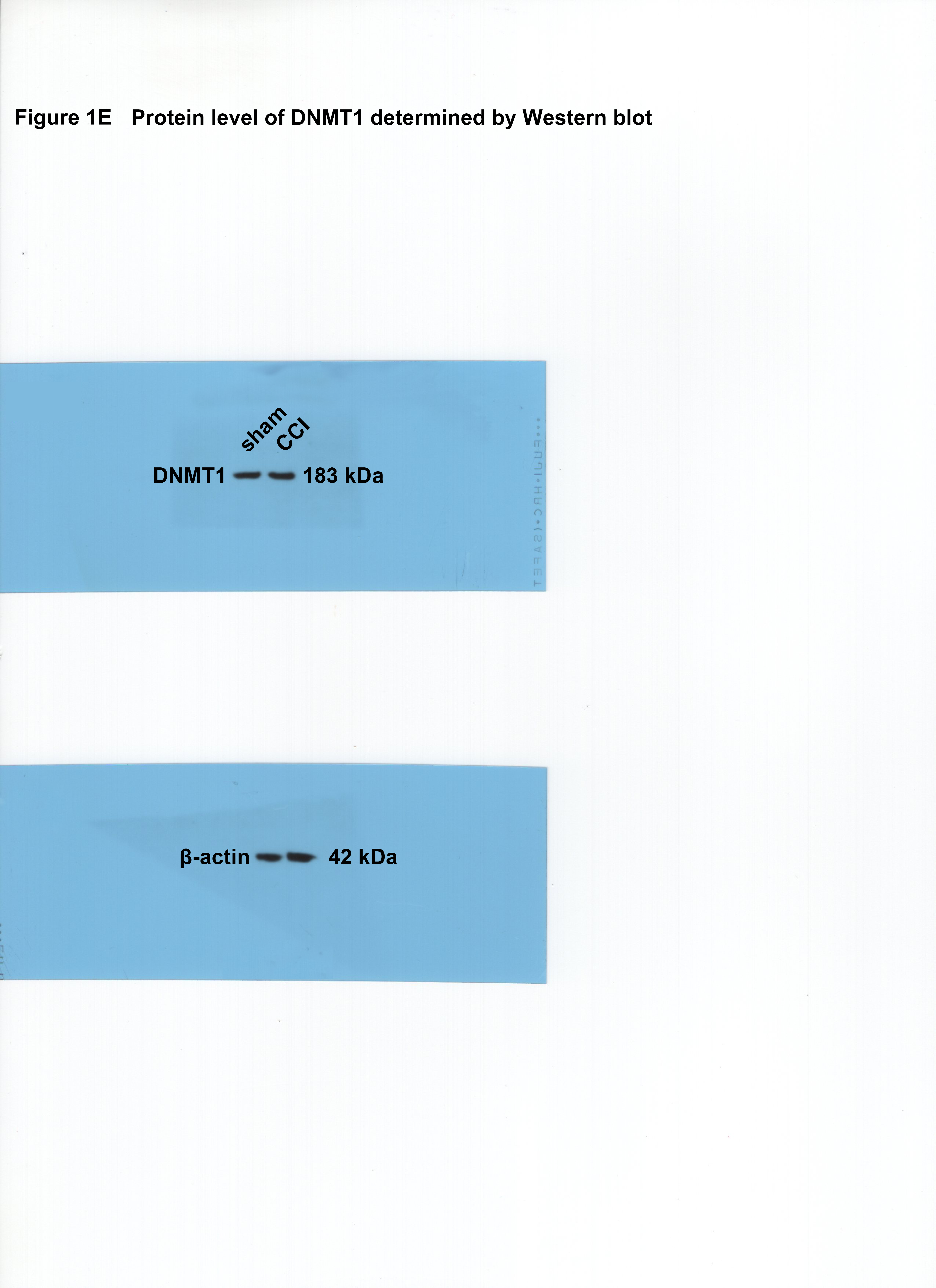

Supplement: Supplementary file 5 — Additional file 5. [file 12883_2022_2860_MOESM5_ESM.tif]

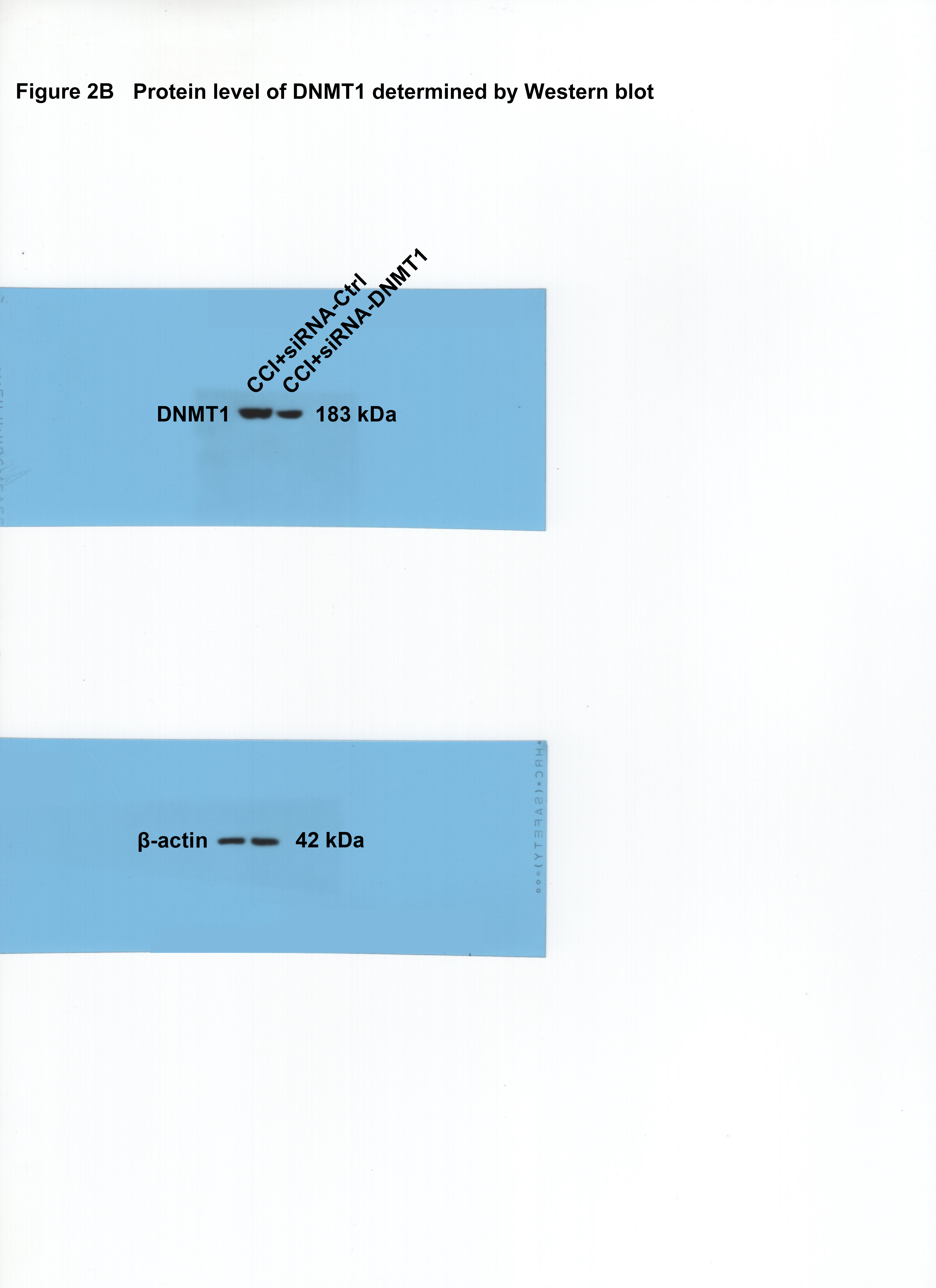

Supplement: Supplementary file 6 — Additional file 6. [file 12883_2022_2860_MOESM6_ESM.tif]

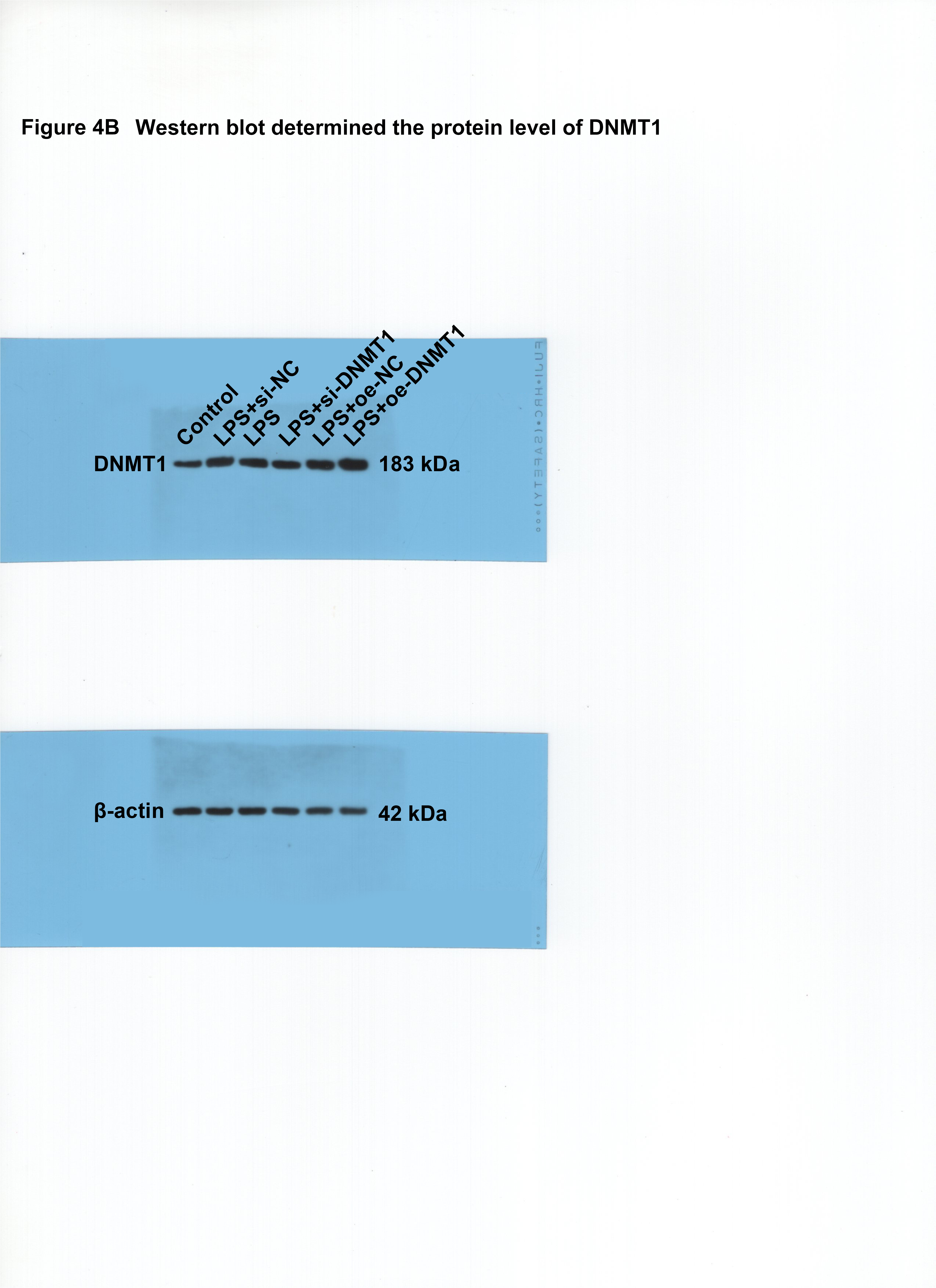

Supplement: Supplementary file 7 — Additional file 7. [file 12883_2022_2860_MOESM7_ESM.tif]

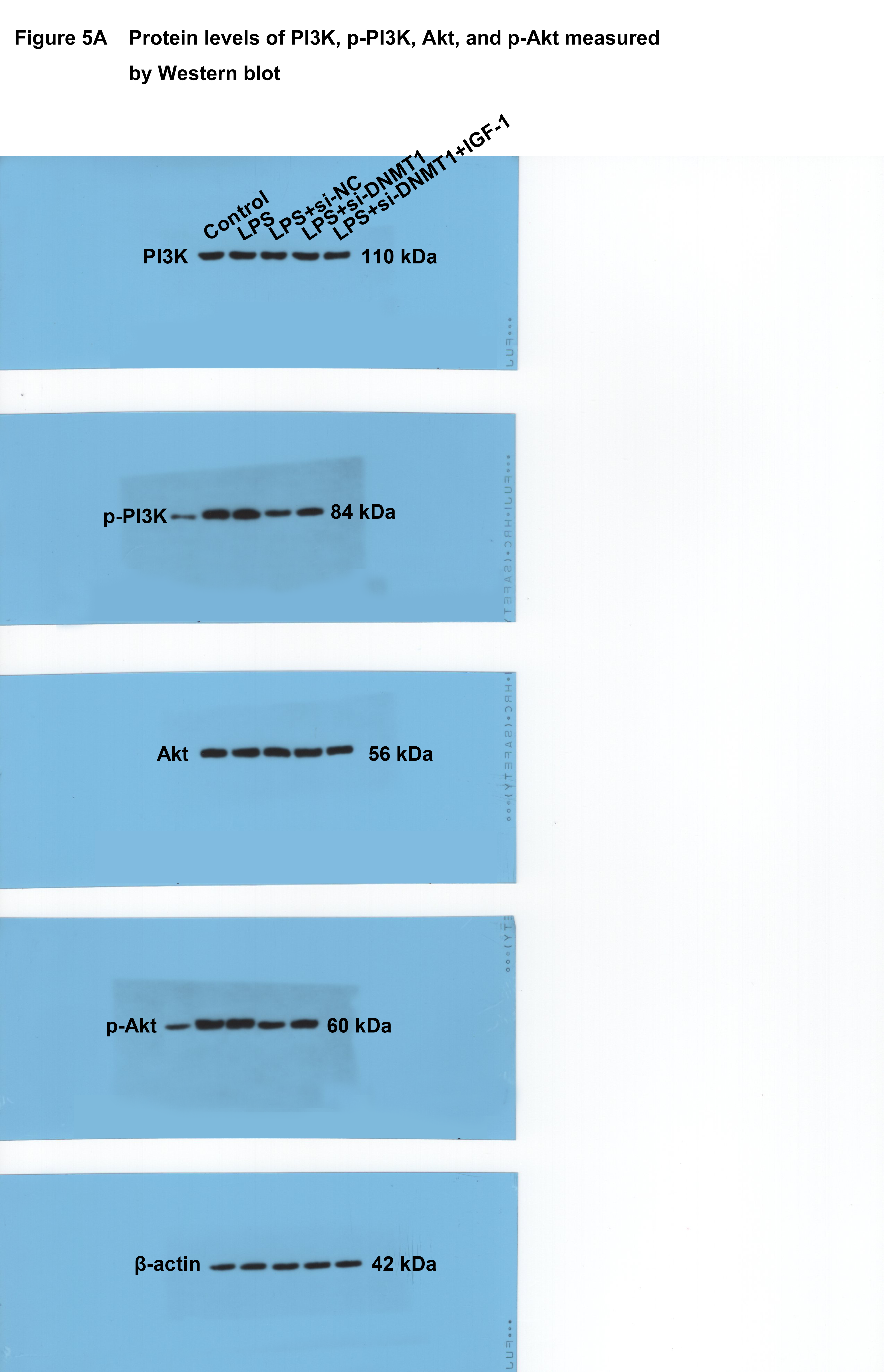

Supplement: Supplementary file 8 — Additional file 8. [file 12883_2022_2860_MOESM8_ESM.tif]

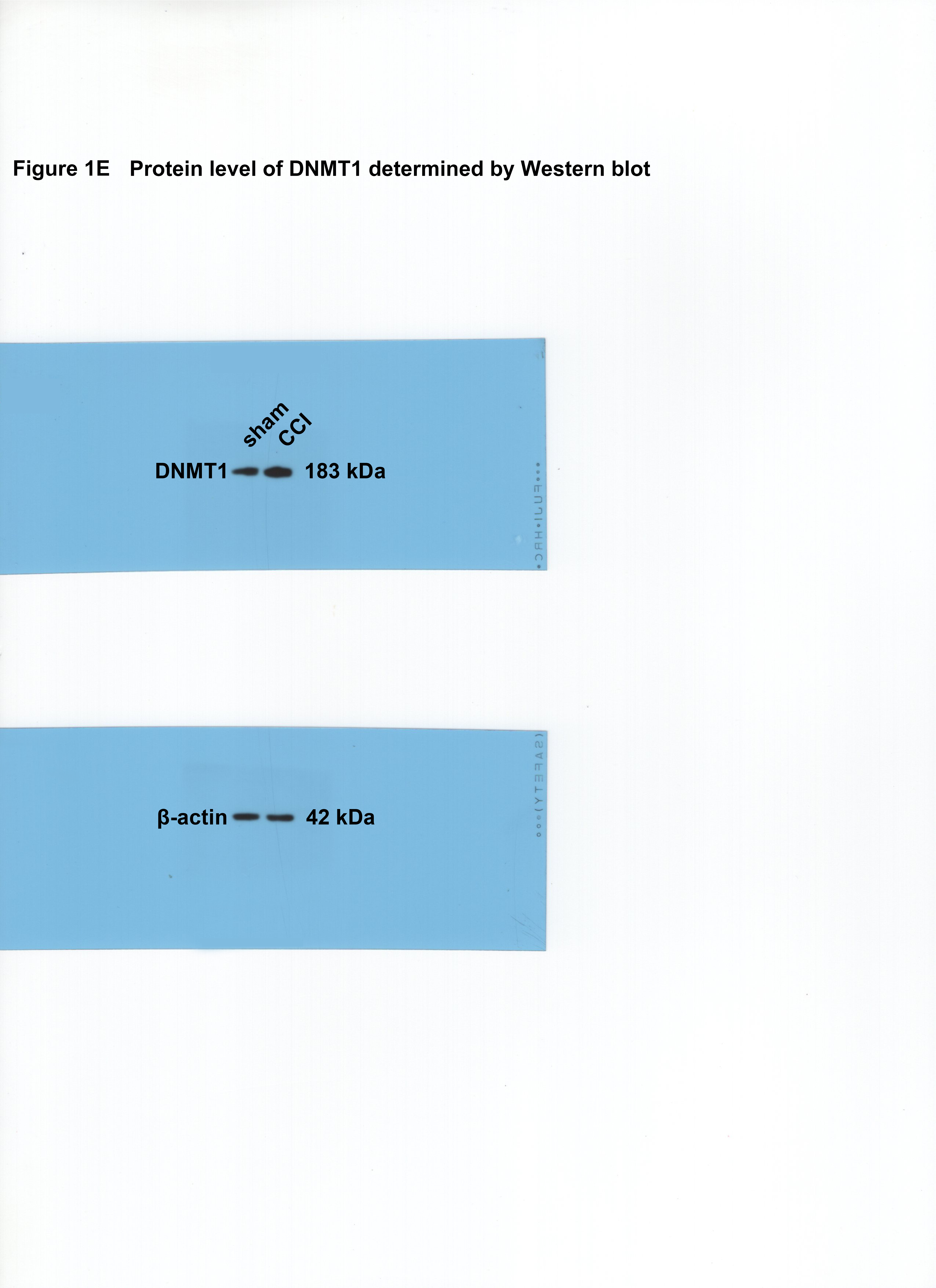

Supplement: Supplementary file 9 — Additional file 9. [file 12883_2022_2860_MOESM9_ESM.tif]

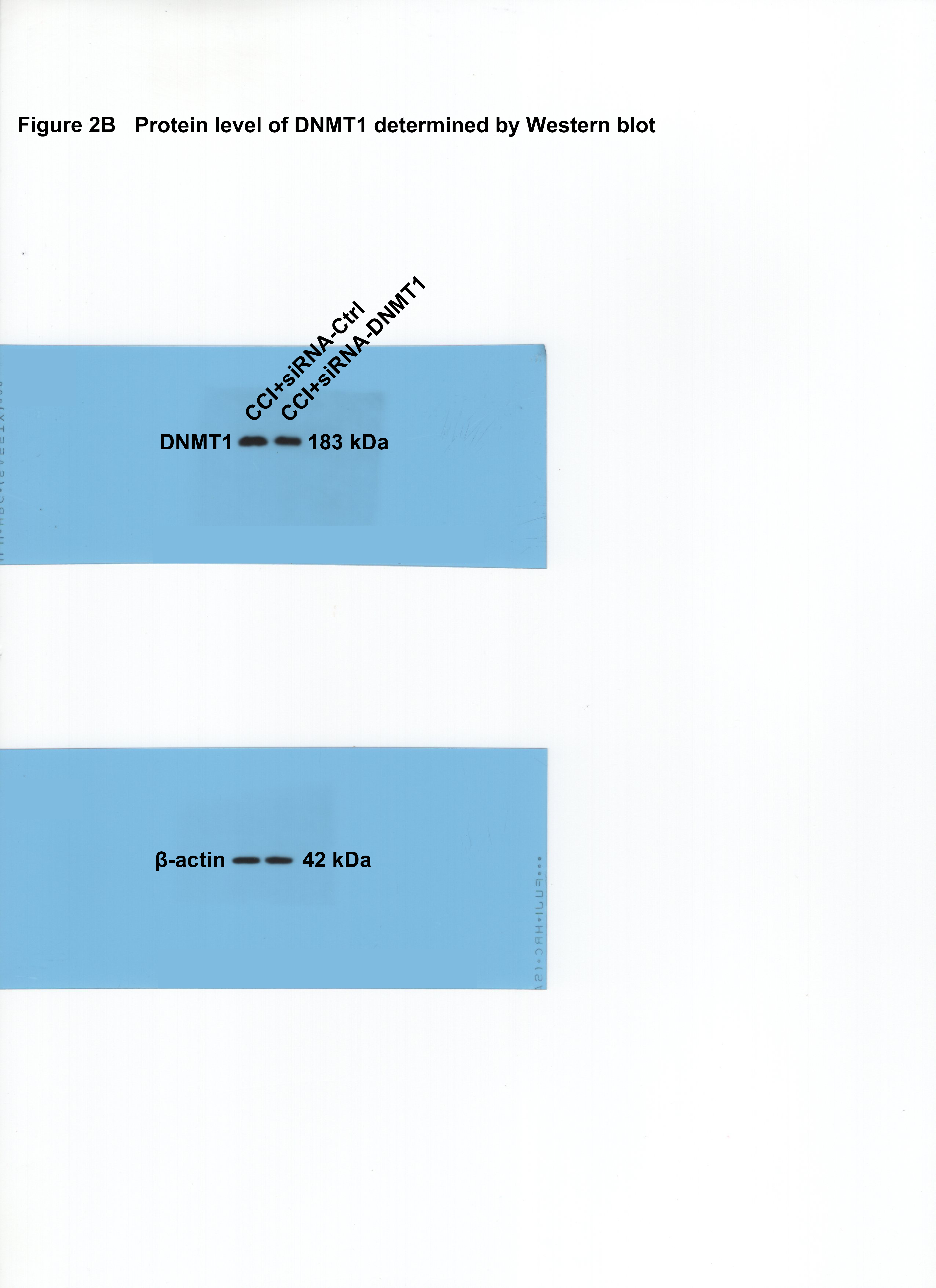

Supplement: Supplementary file 10 — Additional file 10. [file 12883_2022_2860_MOESM10_ESM.tif]

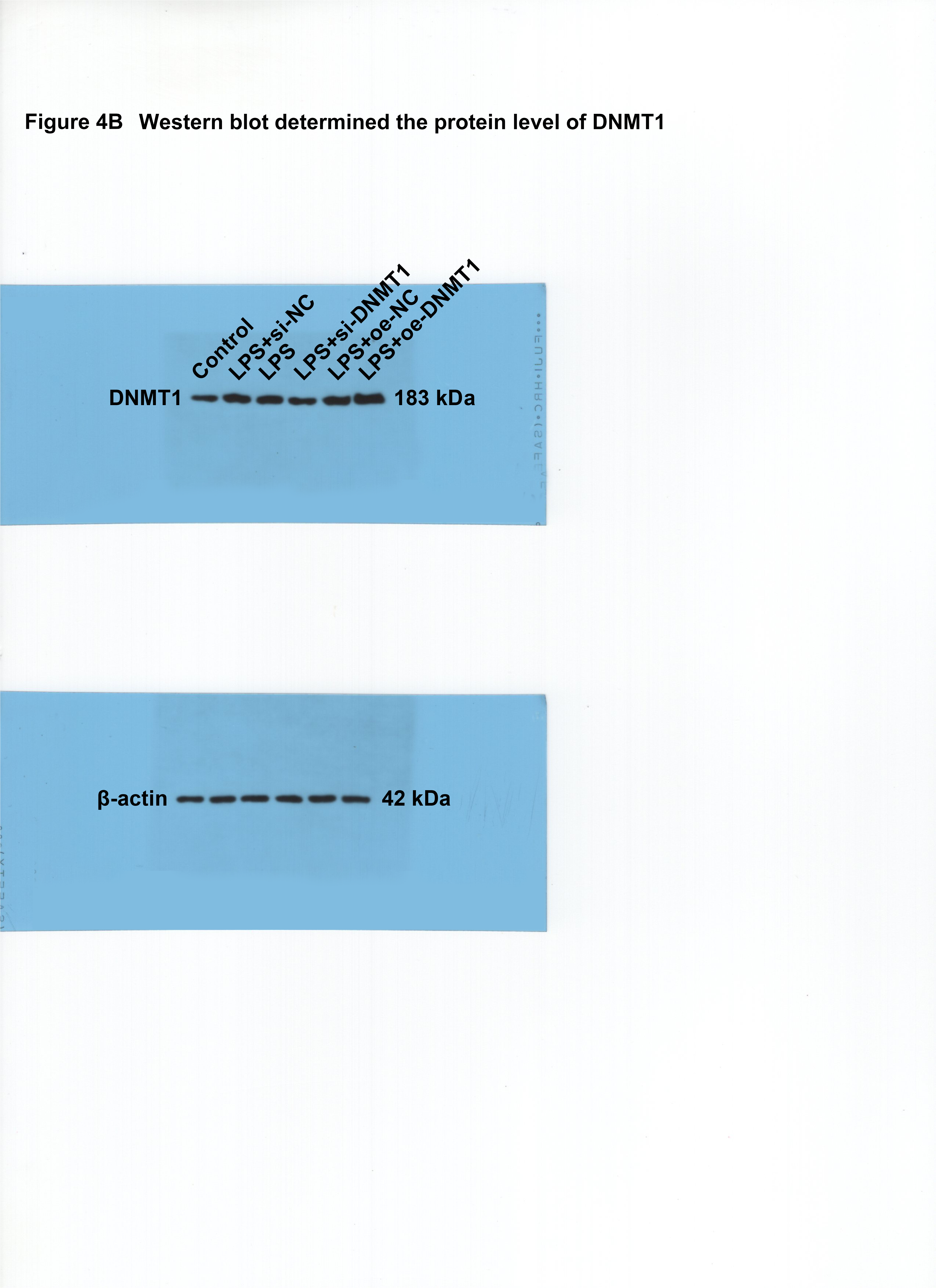

Supplement: Supplementary file 11 — Additional file 11. [file 12883_2022_2860_MOESM11_ESM.tif]

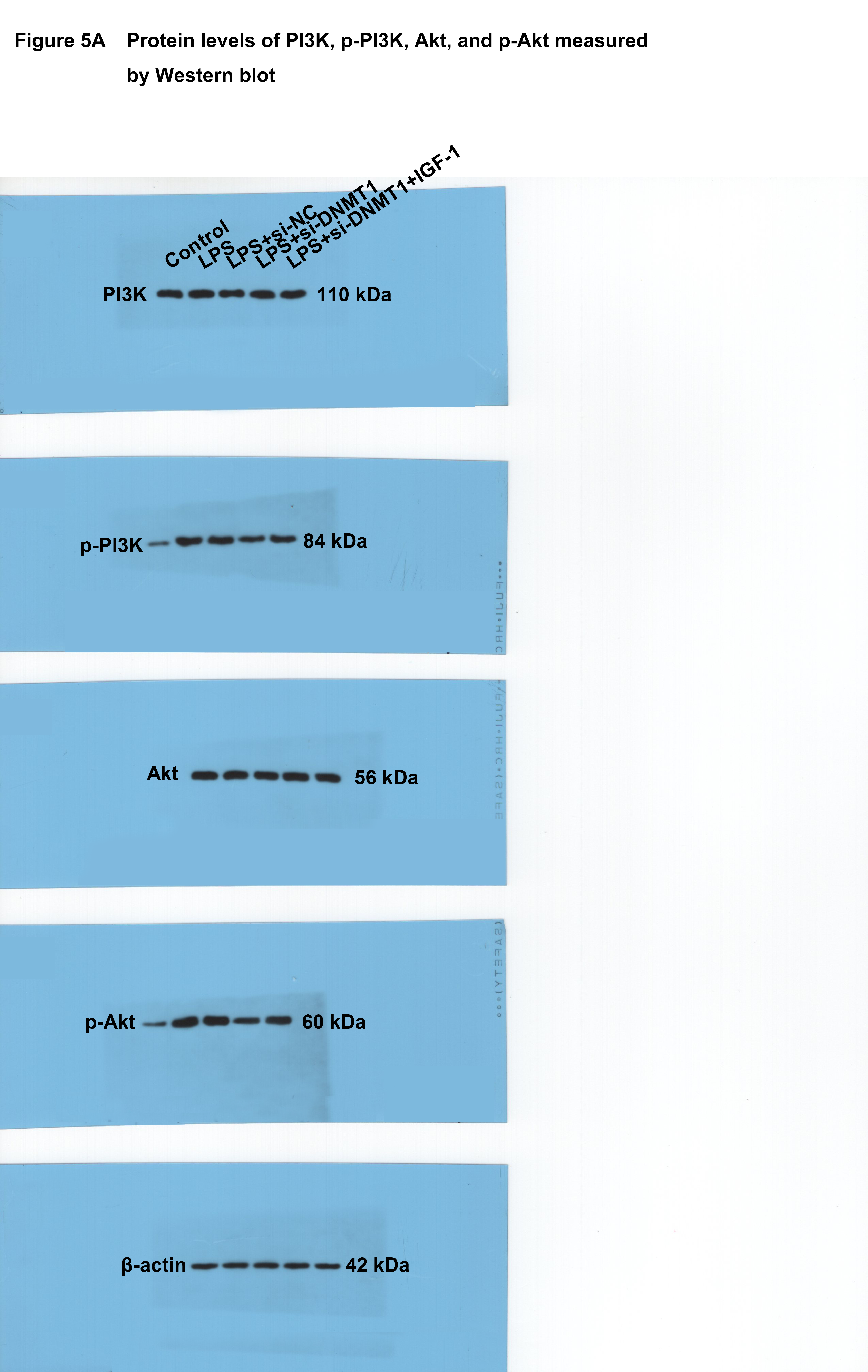

Supplement: Supplementary file 12 — Additional file 12. [file 12883_2022_2860_MOESM12_ESM.tif]
